# A Substituted Diphenyl Amide Based Novel Scaffold Inhibits *Staphylococcus aureus* Virulence in a *Galleria mellonella* Infection Model

**DOI:** 10.3389/fmicb.2021.723133

**Published:** 2021-10-05

**Authors:** Biswajit Mishra, Rajamohammed Khader, Lewis Oscar Felix, Marissa Frate, Eleftherios Mylonakis, Susan Meschwitz, Beth Burgwyn Fuchs

**Affiliations:** ^1^Division of Infectious Diseases, Rhode Island Hospital, Alpert Medical School of Brown University, Providence, RI, United States; ^2^Department of Chemistry, Salve Regina University, Newport, RI, United States

**Keywords:** antimicrobial compounds, drug discovery, *Galleria mellonella*, quorum sensing antagonist, *Staphylococcus aureus*, virulence

## Abstract

Antimicrobial compounds can combat microbes through modulating host immune defense, inhibiting bacteria survival and growth, or through impeding or inhibiting virulence factors. In the present study, a panel of substituted diphenyl amide compounds previously found to disrupt bacterial quorum sensing were investigated and several were found to promote survival in the *Galleria mellonella* model when provided therapeutically to treat a Gram-positive bacterial infection from methicillin-resistant *Staphylococcus aureus* strain MW2. Out of 21 tested compounds, *N*-4-Methoxyphenyl-3-(4-methoxyphenyl)-propanamide (AMI 82B) was the most potent at disrupting *S*. *aureus* virulence and promoted 50% larvae survival at 120 and 96 h when delivered at 0.5 and 5 mg/Kg, respectively, compared to untreated controls (*p* < 0.0001). AMI 82B did not exhibit *G*. *mellonella* toxicity (*LC*_50_ > 144 h) at a delivery concentration up to 5 mg/Kg. Further assessment with mammalian cells suggest AMI 82B hemolytic effects against erythrocytes has an *HL*_50_ greater than the highest tested concentration of 64 μg/mL. Against HepG2 hepatic cells, AMI 82B demonstrated an *LD*_50_ greater than 64 μg/mL. AMI 82B lacked direct bacteria inhibition with a minimal inhibitory concentration that exceeds 64 μg/mL and no significant reduction in *S*. *aureus* growth curve at the same concentration. Assessment via qPCR revealed that AMI 82B significantly depressed quorum sensing genes *agr*, *spa*, and *icaA* (*p* < 0.05). Thus, AMI 82B therapeutic effect against *S*. *aureus* in the *G*. *mellonella* infection model is likely an influence on bacterial quorum sensing driven virulence factors and provides an interesting hit compound for this medically important pathogen.

## Introduction

**FIGURE 1:**
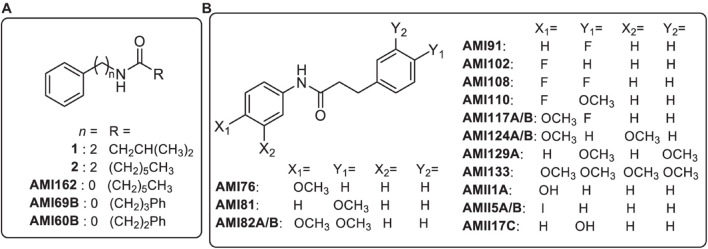
Chemical structures of marine bacterial secondary metabolites (**1** and **2**) and synthetic analogs. Panel **(A)** analogs represent modifications to the acyl group (R), and distance of the amide bond from the phenyl group (*n*). Panel **(B)** analogs include substitutions on the phenyl rings (X and Y) of analog AMI60B.

Bacteria use quorum sensing (QS) to aid in pathogenesis through the production of virulence factors necessary for infection or evading host defenses through the production of a protective biofilm matrix to impede immune exposure (Roilides et al., 2015). Previously, Meschwitz et al. (2019) reported the synthesis of a panel of phenethylamide compounds that target QS-controlled bacteria. The panel of 21 compounds (Figure 1) are the result of a structure-activity relationship (SAR) study undertaken to determine the effect of altering several structural features of the phenethylamide secondary metabolites, **1** and **2**, produced by the marine bacteria, *Halobacillus salinus* and *Vibrio neptunius*, respectively, and shown to inhibit QS controlled phenotypes in multiple Gram-negative reporter strains (Teasdale et al., 2009). Initially, the panel of analogs was designed to explore the effects of modifications to the acyl group (R), and distance of the amide bond from the phenyl group (*n*) (Figure 1, panel A). Reducing *n* to zero and incorporation of an additional phenyl ring on the acyl side chain (R) resulted in the most active analogs. Further modifications retained this structural motif and included substitutions on the phenyl rings (X and Y) (Figure 1, panel B). Previous work on diphenyl beta-keto esters as QSIs of *Vibrio harveyi* BB120, by members of our group, showed the most active derivatives to be 4-fluoro and 4-methoxy phenyl substituted analogs (Forschner-Dancause et al., 2016).

The majority of the synthesized compounds demonstrated inhibition of QS-regulated phenotypes in biosensor strains, including bioluminescence in *V. harveyi* and purple pigment production in *Chromobacterium violaceum* (Meschwitz et al., 2019). Many of the compounds also inhibited the production of green fluorescent protein (GFP) in assays with the mutant *Escherichia coli* biosensor JB525, which contains the *lux*R–P_luxI_ from *Vibrio fischeri* fused to *gfp*, suggesting that the active analogs interact with the Lux-R-type proteins to inhibit QS (Manefield et al., 1999; Andersen et al., 2001). Building on these findings, we examined the impact to virulence of a Gram-postive bacteria, *Staphylococcus aureus*.

Present in both Gram-positive and negative bacteria, QS is regulated through population densities that ultimately influence cells via chemical signaling (Rutherford and Bassler, 2012). Small diffusible signal molecules called auto-inducers facilitate bacterial communication (Miller and Bassler, 2001). Hence, the ubiquitous presence of the conserved QS systems makes this an attractive drug target to counteract biofilms or microbial virulence.

The QS system was first characterized in *V. fischeri* which served as a robust model for the design and development of QS antagonist compounds (Nealson and Hastings, 1979). The basic mechanics of this system are conserved in other bacteria, comprised of five structural genes known as *luxCDABE* with two regulatory genes (luxR and luxI) (Miller and Bassler, 2001). The threshold autoinducer level dictates the LuxR-autoinducer complex binding to the *luxCDABE* promoter, resulting in increased expression of the autoinducer, and a feedback loop is ensured. In *S. aureus*, the *agr* system plays a critical role in virulence gene expression. The *agr* locus is composed of two promotors encoding RNA II (P2) producing *agr* A-D, while the other P3 promotor encodes RNAIII that makes only delta-hemolysin. An autoinducing peptide (AIP) produced by AgrD and B works as the primary QS signal (Novick et al., 1995). Interuption of these signals through compounds antagonizing QS could affect microbial virulence factors and the ability of bacteria to establish an infection.

In the current study, the potential QS antagonist compounds were tested for *in vivo* efficacy in a *G. mellonella-S. aureus* infection model (Fuchs et al., 2016; Tsai et al., 2016) that initially screened for both toxicity and compound efficacy. Toxicity of active compounds was also assessed using erythrocytes and liver-derived HepG2 cells. Finally, target impact was assessed against a panel of *S*. *aureus* QS genes that influence bacterial virulence. The overall interrogation of the compound panel provides insight into efficacy against Gram-positive bacterial virulence using a whole organism infection model.

## Materials and Methods

### Bacterial Strains and Growth Conditions

The bacterial strain used in this study was *S. aureus* MW2 (BAA-1707; ATCC, Manassas, VA, United States), a methicillin-resistant isolate. Bacteria were grown in tryptic soy broth (TSB) (BD, Franklin Lakes, NJ, United States) with agitation at 37°C.

### Quorum Sensing Antagonist Chemical Syntheses

Substituted diphenyl amide compounds were synthesized using a coupling reaction between an aromatic carboxylic acid and a substituted aniline as previously described (Meschwitz et al., 2019). Briefly, the appropriate carboxylic acid (4 mmol, 1 eq) in 50 mL acetonitrile was treated with HB TU [N,N,N′,N′-Tetramethyl-O-(1H-benzotriazol-1-yl)uranium hexafluorophosphate] (1.2 eq), diisopropylethyamine (1.5 eq), and the requisite amine (4 mmol, 1 eq). The reaction was stirred overnight at ambient temperature, concentrated in vacuo, and then partitioned between ethyl acetate and 0.1 M HCl. The organic phase was separated, sequentially washed with saturated sodium bicarbonate and water, dried over anhydrous sodium sulfate, filtered and concentrated in vacuo. The resulting products were purified by either crystallization (ethyl acetate and hexane, compounds labeled A) or automated column chromatography on silica (compounds labeled B, CombiFlash Teledyne Isco, Lincoln, NE, United States) using a linear gradient of hexanes in ethyl acetate (0–100%). Percent yields ranged from 30 to 89%. Compounds were assessed for purity using nuclear magnetic resonance (NMR) spectroscopy and mass spectrometry methods.

### *In vivo* Toxicity of Quorum Sensing Compounds in *G. mellonella*

To investigate the whole organism toxicity and protective effects of the QS compounds, we used the greater wax moth *G. mellonella* (Vanderhorst Wholesale, St. Mary’s, OH, United States) as an infection model (Fuchs et al., 2016; Johnston et al., 2016; Mishra et al., 2018). Groups (*n* = 16, 250–350 mg) consisted of larvae that did not receive any injections, larvae that received PBS (vehicle), and groups injected with investigational compounds interrogated at four concentrations (25, 15, 5, and 0.5 mg/Kg). Vancomycin (25 mg/Kg) was used as a positive control. Larvae received 10 μL of compound using a Hamilton syringe at the last left proleg. The larvae were incubated at 37°C for 6 days and inspected every 24 h for live and dead larvae. The larvae were considered dead if they did not respond to physical touch.

### Therapeutic Evaluations

To assess our collection of compounds at augmenting microbial virulence, the compounds were assessed in the *G*. *mellonella-S. aureus* infection model. *S*. *aureus* MW2 bacteria were grown to exponential phase from an overnight culture then washed three times with PBS and suspended in PBS at OD_600_ ∼ 0.3. Experimental groups consisted of larvae that did not receive any injection, insects that were provided PBS (vehicle), larvae infected with bacteria only, or bacteria followed by investigational compounds (5 and 0.5 mg/Kg). Vancomycin (25 mg/Kg) was used as a positive control. Ten μl (2 × 10^6^ cells/mL) of the prepared bacteria was injected in the respective group, followed by the investigational compounds after 1 h (10 μL volume). Larvae were monitored for 5 days and assessed for survival daily.

### Prophylactic Assessment

In the protection assays, the investigational compounds were provided prior to bacterial infection. Larvae received the compounds (10 μL volume) 1 h ahead of the bacteria injections. Similar groups and doses were maintained as described above to establish a correlation between both the treatment options. Subsequently, the larvae were monitored daily for survival over a span of 5 days.

### Hemolysis of Human Red Blood Cells

When therapeutic agents are injected, one of the first contacts is with erythrocytes. Therefore, it is important to ensure investigational compounds are not exerting hemolysis. The ability of the investigational compounds to cause erythrocyte lysis was evaluated, as described previously (Kim et al., 2018). Human erythrocytes were purchased from Rockland Immunochemicals (Limerick, PA, United States), washed 3 times in equal volume of PBS, and suspended as 4% human red blood cells (hRBCs) in PBS. Fifty μL of the blood cells were added to 50 μL of the investigational compound solution in PBS serially diluted from 64 to 1 μg/ml in a 96-well microtiter plate. Triton-X 100 and PBS were used as positive and negative controls. Upon adding the blood cells, the plate was incubated at 37°C for 1 h and then centrifuged at 500 × *g* for 5 min. Fifty μL of the supernatant was transferred to a fresh 96-well plate, and absorbance was read at room temperature using a spectrophotometer (SpectraMax M2, Molecular Devices, San Jose, CA, United States) at 540 nm. Percent hemolysis was calculated considering 100% hemolysis caused by 1% Triton X-100, and 0% incurred from PBS. Values were represented as the mean of duplicates. The following formula calculated the percentage of hemolysis:

% HEMOLYSIS = {(Abs540 nm in the QS compounds solution-Abs540 nm in PBS)/(Abs540 nm in 0.1% Triton X-100-Abs540 nm in PBS)} × 100.

### Hepatotoxicity Assay

Since compounds often pass through the liver, it is important to ensure investigational molecules do not induce liver cell death at concentrations that also inhibit bacterial virulence. Therefore, hepatotoxicity was invested using a liver cell line. HepG2 cells were used as a model to initially assess mammalian cell cytotoxicity using methods described previously (Kim et al., 2018) with minor modifications. Briefly, cells were grown in Dulbecco’s Modified Eagle Medium (DMEM) (Gibco, Thermofisher Scientific, Waltham, MA, United States) supplemented with 10% fetal bovine serum (FBS) (Gibco, Thermofisher Scientific, Waltham, MA, United States) and 1% penicillin/streptomycin (Gibco, Thermofisher Scientific, Waltham, MA, United States) and maintained at 37°C with 5% CO_2_. Cells were harvested and resuspended in fresh medium, and 100 μL were distributed in a 96-well plate at 5 × 10^4^ cells/well. Compounds were serially diluted in serum and antibiotic-free DMEM added to the monolayer of the cells, and the plates were incubated at 37°C with 5% CO_2_ for 24 h. At 4 h, before the end of the incubation period, 10 μL of 2-(4-iodophenyl)-3-(4-nitrophenyl)-5-(2,4-disulfophenyl)-2H-tetrazolium (WST-1) solution (Roche, Mannheim, Germany) was added to each well. WST-1 reduction was monitored at 450 nm using a spectrophotometer (SpectraMax M3, Molecular Devices, San Jose, CA, United States). Assays were performed in triplicate, and the percentage of cell survival was calculated.

### Inhibition of *S. aureus* Biofilm Formation

The effectiveness of the investigational compounds to inhibit biofilm formation was evaluated by following an established protocol with minor modifications (Liu et al., 2018; Mishra et al., 2018). In brief, exponential cultures of *S. aureus* MW2 were prepared in TSB from overnight inoculated cultures. 50 μL of bacterial suspension adjusted to (OD_600_ ∼ 0.03) in fresh TSB were incubated with 50 μL of serially diluted 2× compounds solution in a flat-bottomed 96-well polystyrene microtiter plates (Corning Costar Cat No. 3595, Thermofisher Scientific, Waltham, MA, United States) for 24 h at 37°C without shaking. Bacteria without drug treatment were considered negative control and represented 100% growth. After incubation, the media was carefully pipetted out, and the wells were washed with PBS (Gibco, Thermofisher Scientific, Waltham, MA, United States) to remove planktonic cells followed by the addition of XTT [2,3-bis(2-methyloxy-4-nitro-5-sulfophenyl)-2H-tertazolium-5-carboxanilide]. Quantitation of biofilm attachment followed the manufacture’s instructions with minor adjustments (ATCC, Manassas, VA, United States). The calorimetric intensity of the XTT dye at 450 nm was measured by the SpectraMax M2 Multi-mode Microplate Reader (Molecular Devices, San Jose, CA, United States). The percentage of biofilm growth was plotted compared to negative control wells without treatment.

### Prevention of Static Biofilm Attachment

Static *S. aureus* biofilms were made on 96-well microtiter plates following an established method with slight modification (Mishra and Wang, 2017; Liu et al., 2018). Briefly, 180 μL of overnight bacterial culture in TSB broth supplemented with 1% glucose were mixed with 20 μL of serially diluted compounds and incubated at 37°C for 1 h without shaking. After aspirating the planktonic cells, residual adhesive cells in each well were washed with PBS (Gibco, Thermofisher Scientific, Waltham, MA, United States; pH 7.2) to remove non-attached cells. Subsequently, 200 μl of 99% methanol was added for fixation and the plates were allowed to sit for 15 min. The plates were finally aspirated and dried. Lastly, cells were stained with 200 μL of 1% crystal violet in water for 5 min. Excess stain was gently rinsed off with tap water, and plates were air-dried. The stain was solubilized in 200 μl of 33% glacial acetic acid to allow colorimetric measurement at 595 nm using a SpectraMax M2Multi-mode Microplate Reader (SpectraMax M2, Molecular Devices, San Jose, CA, United States).

### Quantitative Polymerase Chain Reaction

*Staphylococcus aureus* MW2 culture was grown overnight in TSB to evaluate the effect of investigational compounds on bacterial QS genes that contribute to bacterial virulence. Cells were cultured to the exponential phase and harvested when the OD_600_ reached ∼0.4. The cells were washed with PBS and exposed to 4 μg/mL concentration of the test compounds for 24 h. RNA was isolated using the RNeasy mini kit (Qiagen, Hilden, Germany) based on the manufacturer’s instructions. cDNA synthesis and quantitative reverse transcription (RT)-PCR were carried out as recommended by the manufacturer (Bio-Rad, CA, United States) using the primers listed in Supplementary Table 1. The quantitative polymerase chain reaction (qPCR) cycling conditions were: 95°C for 30 s, 40 cycles at 95°C for 5 s, 55°C for 30 s, and finished with a melt curve analysis from 65 to 95°C.

### Statistics

Kaplan–Meier survival curves were plotted with GraphPad Prism version 8.4.1 (GraphPad Software, La Jolla, CA, United States). Statistical analysis was carried out using the same program, and *p* < 0.05 was considered significant comparing the test group to the PBS treated group. The software uses a log-rank (Mantel-Cox) test to compare the survival curves. For the biofilms and the qPCR analysis, the level of significance was tested using a student *t*-test and *p* values <0.05 were considered significant.

## Results

### Toxicity Assessment in the *G. mellonella* Model

**FIGURE 2:**
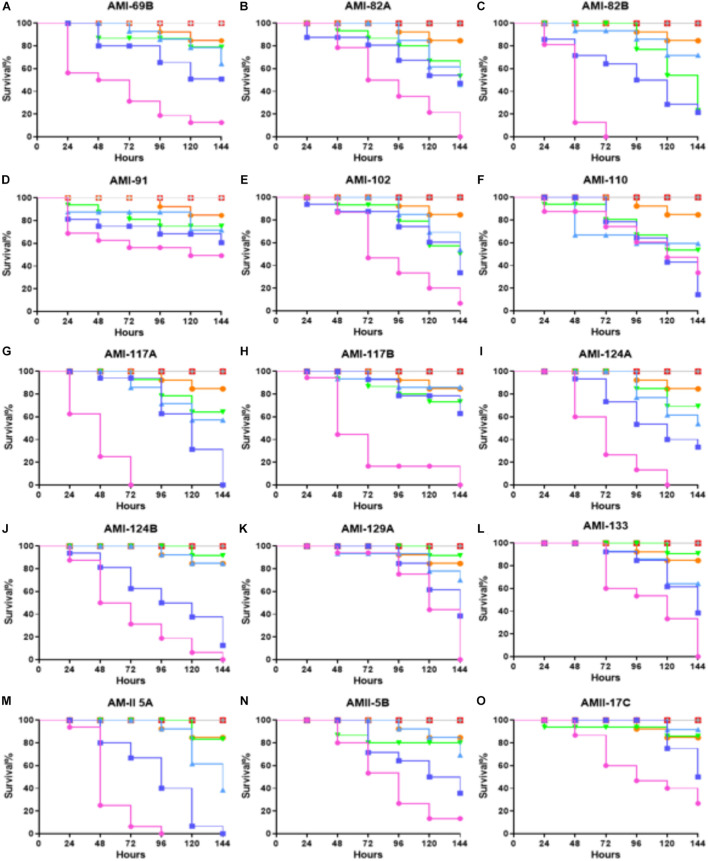
Whole larvae survival assay for toxicity assessment of the test compounds. Plotted here **(A–O)** are the toxicity profiles of selected compounds showing at least *LC*_50_ of >150 h for some portion of the tested concentrations. Legend represents: 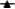; No treatment: 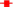; PBS treated: 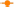; DMSO treated: 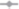; Vancomycin treated at 25 mg/Kg: 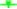; representative compound treated at 0.5 mg/Kg: 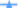; 5 mg/Kg: 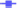; 15 mg/Kg and 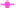; 25 mg/Kg. Kaplan–Meier survival curves were plotted with GraphPad Prism version 6.048.4.1 (GraphPad Software, La Jolla, CA, United States). Statistical analysis was carried out using a log-rank (Mantel-Cox) test comparing the each group to the PBS treated group. *p* value < 0.05 was considered significant.

A panel of 21 investigational compounds (Figure 1) with previous assessment as QS antagonists were screened in a whole organism insect model system using *G*. *mellonella*, a known excellent model for *in vivo* toxicity and efficacy testing (Johnston et al., 2016). All compounds were injected with a concentration of 25, 15, 5, or 0.5 mg/Kg with respect to the larvae body weight, and the toxicity profiles were recorded for 144 h (Figure 2 and Supplementary Figure 1). It is interesting to note that 15 compounds demonstrated a lethal concentration (LC)_50_ of >144 h at least for the two lowest tested concentrations of 0.5 and 5 mg/Kg (Figure 2).

Among the least toxic conpounds were AMI 69B, AMI 82A, AMI 82B, AMI 91, AMI 102, AMI 110, AMI 117A, AMI 117B, AMI 124A, AMI 124B, AMI 129A, AMI 133, AMII 5B, and AMII 17C. However, a few compounds from this sub-group even showed reduced toxicities, AMI 69B, AMI 82A, AMI 91, AMI 117B, of *LC*_50_ > 144 h for an elevated concentration of 15 mg/Kg. Interestingly, dose-dependence toxicity was not found in these sets of compounds even though they all belong to the same molecular scaffold structurally.

Non-dose dependent efficacy profiles were observed for AMI 60B, AMII 1A, AMII 5B, and AMII 17C. The rest of the compounds followed the dose-dependent toxicity curves. Compounds AMI 82A, AMI 82B, AMI 91, AMI 117B, AMI 124A, AMI 129A, AMI 133, and AMII 17C were observed for very low toxicity. For the next set of efficacy experiments, the least toxic compounds (*LC*_50_ survival > 120 h) at the two lowest concentrations: AMI 69B, AMI 82A, AMI 82B, AMI 91, AMI 102, AMI 110, AMI 117A, AMI 117B, AMI 124A, AMI 124B, AMI 129A, AMI 133, AMII 5B, and AMII 17C were selected for further evaluation in order to determine if these compounds could provide therapeutic or prophylactic efficacy in the invertebrate model host *G*. *mellonella* during an *S*. *aureus* infection.

### *In vivo* Efficacy of Investigational Compounds

**FIGURE 3:**
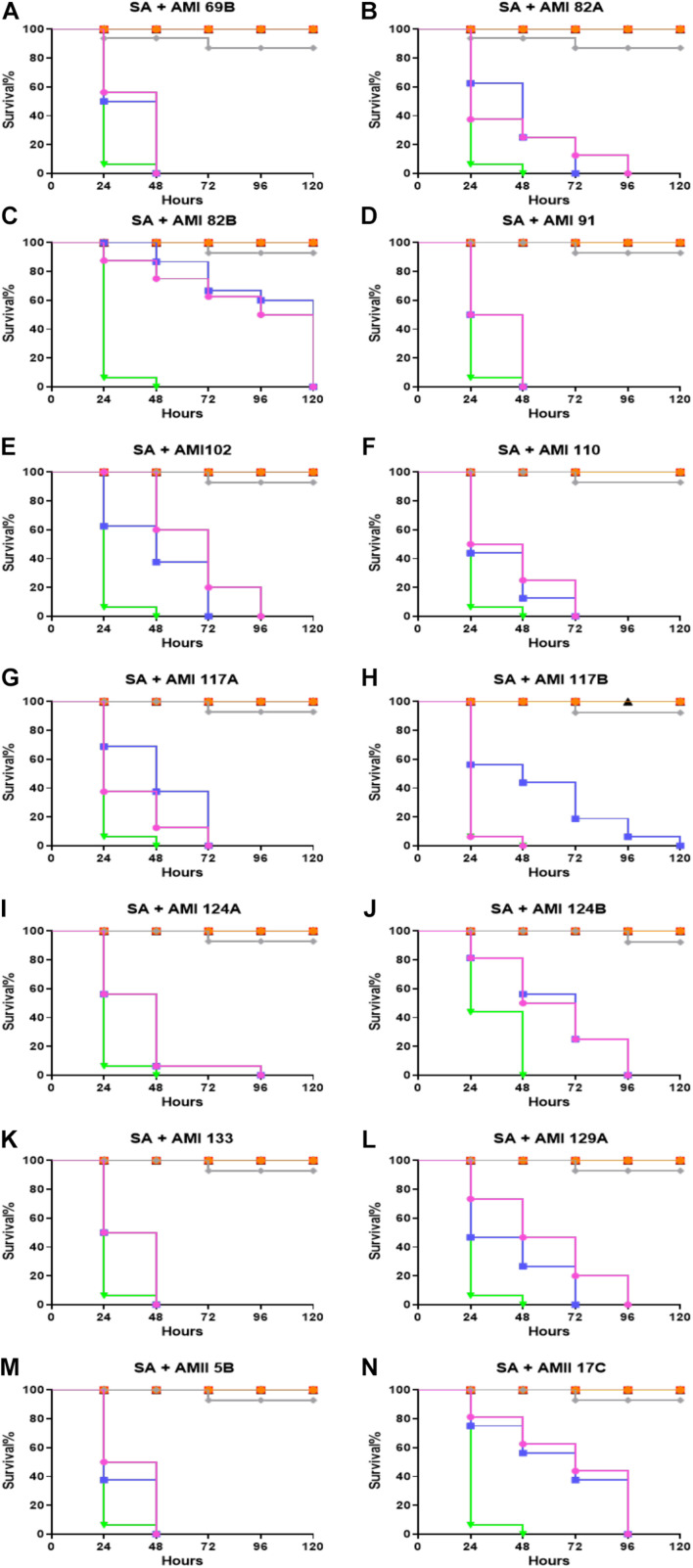
Whole larvae survival assay for efficacy assessment of the test compounds. Plotted here **(A–N)** are the survival profiles of selected compounds. Legend represents: 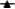 No treatment;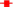 PBS only; 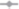 DMSO only; 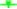 *S. aureus* infected; 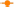 *S. aureus* + Vancomycin treated at 25 mg/Kg; 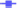 representative compound treated at 0.5 mg/Kg; and 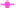 5 mg/Kg. Kaplan–Meier survival curves were plotted with GraphPad Prism version 6.048.4.1 (GraphPad Software, La Jolla, CA, United States). Statistical analysis was carried out using a log-rank (Mantel-Cox) test comparing each group to the PBS treated group. *p* value < 0.05 was considered significant.

The therapeutic efficacy of the test compounds with low toxicity were checked in the *G*. *mellonella-S*. *aureus* infection models. In a therapeutic setting, the larvae were infected by the bacteria first in order to mimic a systemic infection. Once infected, the compound were administered after 1 h, testing two different doses: 0.5 and 5 mg/Kg (Figure 3). The majority of *G*. *mellonella* in the control group (*S. aureus* infection treated with PBS only injections) were predominantly dead at 24 h (>90%) with full group death by 48 h. Vancomycin (25 mg/Kg) was used as a positive control and rescued >90% of the larvae for 120 h.

Compounds AMI 69B, AMI 91A, AMI 133, and AMII 5B significantly improve survival at any of the tested concentrations. For these molecules, the treated larvae were dead within 48 h. Fifty percent larval survival for AMI 102 was achieved by 48 and 72 h for 0.5 and 5 mg/Kg, respectively. AMI 129A exhibited 50% survival at approximately 24 h and 48 h for the two tested concentrations. Although the 50% larval survival for AMI 117B was reached at 48 h and 24 h at 0.5 and 5 mg/Kg, larval survival was extended to 120 h at 0.5 mg/Kg. The rest of the investigational compounds did not show differences between the tested concentrations. At the lowest 0.5 mg/Kg dose, 50% larval survival for AMI 82A, AMI 110, and AMI 129A occurred at 24 h. For AMI 117A, AMI 117B, and AMI 124A 50% survival was recorded at 48 h, and for AMI 124B and AMII 17C it was reached at 72 h. Of all the compounds tested, AMI 82B was found to be the most potent and exhibited 50% larvae survival at 120 and 96 h for 0.5 and 5 mg/Kg, respectively (*p* < 0.0001).

**FIGURE 4:**
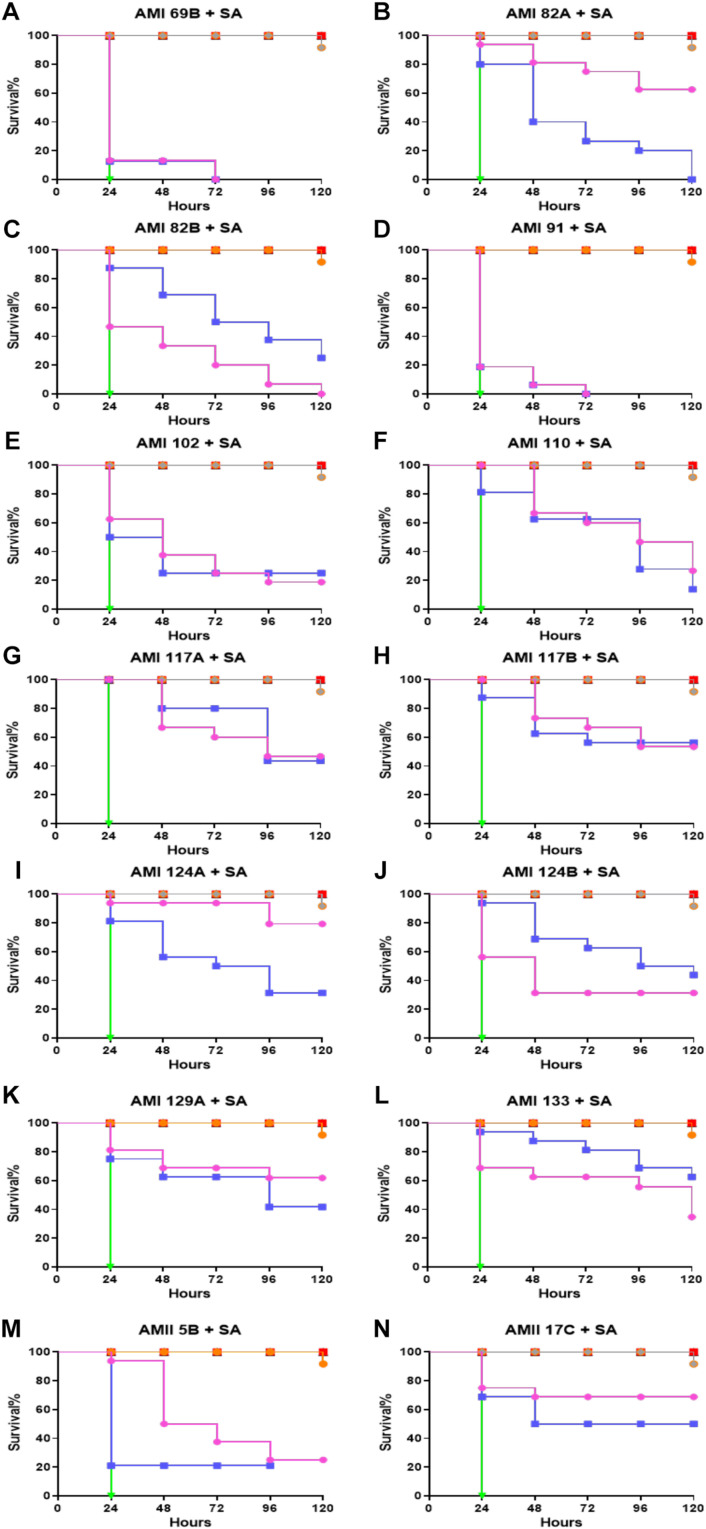
Whole larvae survival assay for protection assessment of the investigational compounds. Plotted here **(A–N)** are the survival profiles of selected compounds. Legend represents: 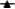 No treatment; 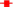 PBS only; 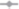 DMSO only; 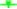 *S. aureus* infected;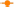 *S. aureus* + Vancomycin treated at 25 mg/Kg; 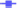 representative compound treated at 0.5 mg/Kg; and 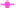 5 mg/Kg. Kaplan–Meier survival curves were plotted with GraphPad Prism version 6.048.4.1 (GraphPad Software, La Jolla, CA, United States). Statistical analysis was carried out using a log-rank (Mantel-Cox) test comparing the each group to the PBS treated group. *p* value < 0.05 was considered significant.

Compounds were assessed to determine potential to provide protection against *S*. *aureus* infection when delivered as prophylaxis (Figure 4). In this assay, the larvae were pretreated with the same set of QS compounds as described above when treating therapeutically deployed compounds during *S. aureus* infection. A similar concentration of the QS compounds (0.5 and 5 mg/Kg) and bacteria were used to learn if there was a prophylactic delivery impact. The group infected with bacteria that did not receive any intervention treatment were all dead within 24 h. The positive control, vancomycin treated group, exhibited 90% larvae survival at 120 h post-infection.

Interestingly, in this assay, all the compounds showed protection of the larvae to bacterial infection. For the least active compounds, the 50% larval survival for AMI 69B, AMI 91A, AMI 102 were found to be 24 h for both 0.5 and 5 mg/Kg except for a small improvement to AMI 102 injected larvae which extended survival to 48 h when provided a 5 mg/Kg dose. Dose dependencies were observed for AMI 82A, AMI 102, AMI 124A, and AMI 129A. At the highest dose of 5 mg/Kg the 50% larval survival rate for AMI 82A, AMI 124A, and AMI 129A was recorded at 120 h (*p* < 0.0001). While in compounds AMI 69B, AMI 91A, AMI 110, AMI 117A, AMI 117B, and AMI 133 there was no difference observed between the doses. In this group, 50% larval survival rate was observed for AMI 117B and AMI 133 at 120 h, followed by 96 h for AMI 110 and AMI 117A (*p* < 0.0001). For compounds AMI 82B and AMI 124B, the lower dose seemed more effective. Additionally, at the 120 h time point, the larval survival rate was recorded for AMI 124A, AMI 129A, AMI 82A, and AMI 117B as 80, 62.5, 62.5, and 52.5%, respectively, at 5 mg/Kg dose (*p* < 0.0001). For lower dose of 0.5 mg/Kg, compounds like AMI 117B and AMI 133 were shown to protect 57.5 and 62.5% of the larvae, respectively, at 120 h (*p* < 0.0001).

### Antimicrobial Activity

The compounds were tested for minimal inhibitory concentrations using a broth microdilution assay following the protocol described in the CLSI guidelines (CLSI, 2015). The compounds were tested over the concentration range of 64–0.0625 μg/mL and were found not to exhibit any antimicrobial activity against *S. aureus* (data not shown). Therefore, prolonged *G. mellonella* survival appears to be a result of influencing bacterial virulence and not influencing growth or directly killing *S*. *aureus* (Supplementary Figure 2).

### Hemolytic Effects of Quorum Sensing Compounds

**FIGURE 5:**
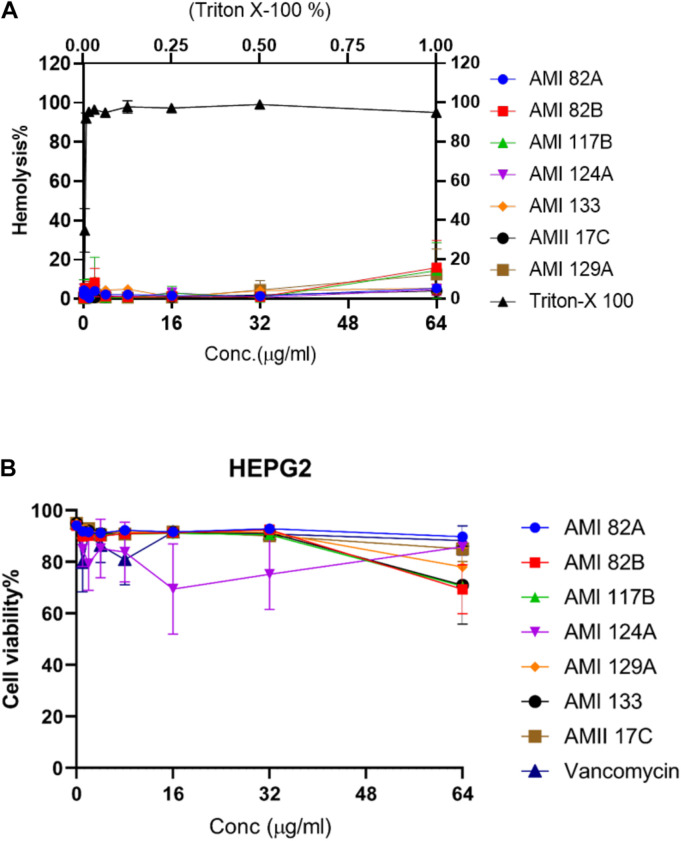
Cytotoxicity for lead compounds. Hemolysis **(A)** was tested in human erythrocytes and viability **(B)** was assessed with HepG2 cells.

Seven of the investigational compounds were selected for further assessment based on exhibiting *in vivo S*. *aureus* inhibitory potential that reflected toxicity *G*. *mellonella LD*_50_ that exceeded 120 h at the 0.5 and 5 mg/Kg in addition to prophylactic or therapeutic potential that promoted larval survival greater than 50% at 120 and 96 h, respectively. Thus, providing the grouping of AMI 82A, AMI 82B, AMI 117B, AMI 124A, AMI 129A, AMI 133, and AMII 17C for additional scrutiny. Antimicrobial compounds are often injected to treat systemic infections so investigational compounds are assessed for hemolytic potential. In this case, the prioritized compounds were assessed for hemolytic potential against 2% human RBCs (Figure 5A).

Interestingly, at the highest concentration of 64 μg/mL, none of the compounds were observed to induce hemolysis. AMI 82B, AMI 117B, and AMI 129A resulted in 10–18% hemolysis and the rest of the compounds including AMI 82A, AMI 124A, AMI 133, and AMII 17C exhibited <10% hemolysis. The positive control, Triton X-100 was 100% hemolytic at 1% concentration. Numerically, the *HL*_50_ (50% hemolysis) for all the tested test compound was assigned as >64 μg/mL.

### Hepatic Cell Toxicity Potential of Investigational Compounds

Since therapeutic compounds are often processed by the liver, toxicity to liver cells is also important. The same set of the selected compounds were tested against the liver-derived HepG2 cell line (Figure 5B) for hepatotoxicity. Excitingly, none of the compounds were found to be highly toxic. Compounds AMI 82B, AMI 117B, and AMI 133 contributed to decreased cell viability around 30%, AMI 129A around 20% and the rest of the tested compounds which included AMI 82A, AMI 124A, and AMII 17C inhibited HepG2 growth by <10%. Thus, the *LD*_50_ for all the tested compounds was >64 μg/mL.

### Inhibition of Biofilms

**FIGURE 6:**
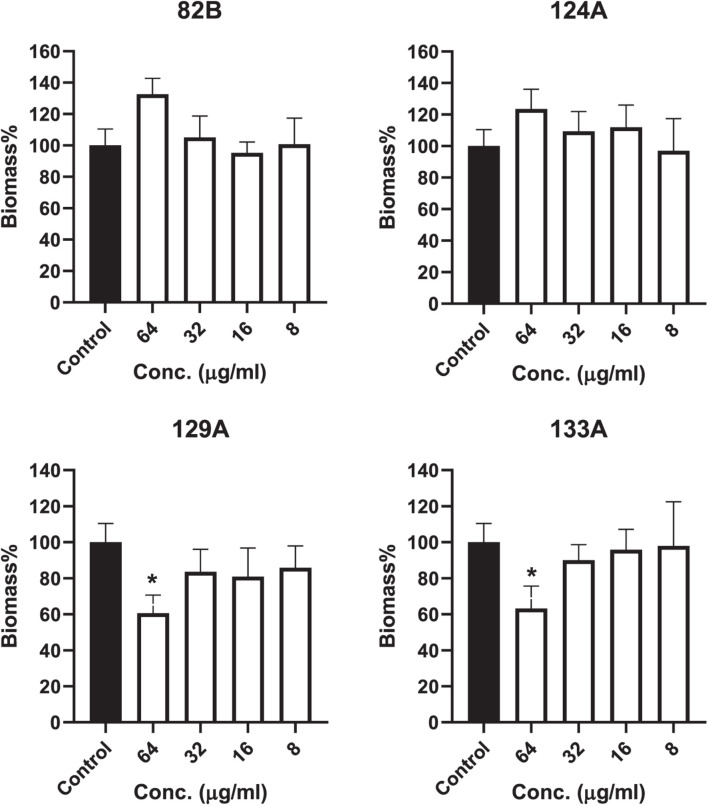
Compound influence on biofilms were assessed. Biofilm biomass was determined by crystal violet staining. (*) refers to significant values with *p* < 0.05 based on a student *t*-test comparing the biofilms treated with respective compounds to the control biofilms (incubated with PBS only).

The investigational compounds, including AMI 82B, AMI 124A, AMI 129A, and AMI 133, were tested for their potential for inhibiting biofilms. Two separate tests were conducted, one to test the potential of the biofilm formation for 24 h. The other assay was directed to determine compound efficacy at inhibiting cell attachment to the substratum. Interestingly, none of the compounds seemed to affect any long-duration biofilm formation in terms of live cell contents up to the tested concentration of 64 μg/mL (determined by XTT) (Supplementary Figure 3). The compounds even failed to inhibit the attachment of initial bacteria to the plates. Interestingly, AMI129A and AMI 133 reduced the initial biomass accumulation at 64 μg/mL (determined by CV staining) (Figure 6). Approximately 40% biomass reduction was observed for AMI 129A and AMI133 against a high-density *S*. *aureus* MW2 strain. With evidence that compounds prolong survival of *S. aureus* infected *G*. *mellonella* and previous findings that the compounds could be QS antagonistic in other organisms, there is potential that a subset of the compounds interrogated may impact portions of the quorum sensing system to augment virulence independent of biofilm.

### Antivirulence Potential

**FIGURE 7:**
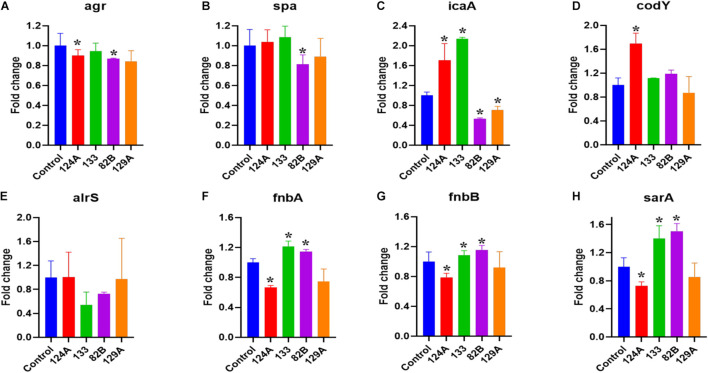
QS genes that contribute to *S. aureus* virulence were assessed with real-time quantitative evaluation (qPCR) by exposing bacteria to lead compounds (at 4 μg/ml). **(A–H)** Major *S. aureus* virulence genes tested. (*) refers to significant values with *p* < 0.05 based on a student *t*-test comparing the compound treated bacterial samples to the control (PBS treated).

A real-time quantitative evaluation of significant genes that play a role in *S. aureus* QS and virulence were tested in the presence of select compounds observed to impact the *G*. *mellonella-S*. *aureus* infection model (Figure 7). The genes included were *agr*, *spa*, *icaA*, *codY*, *alrS*, *fnbA*, *fnbB*, and *sarA*. The bacteria were subjected to 4 μg/mL of AMI 124A, AMI 133, AMI 82B, and AMI 129A for 24 h, giving the bacteria ample time to regulate virulence or QS in their late stationary growth phase. Interestingly, *agr* was down-regulated by a modest but significant amount from AMI 124A and AMI 82B (*p* < 0.05). The compounds AMI 133 and AMI 129A did not seem to affect *agr*. Only AMI 82 B decreased *spa* expression, causing a 20% reduction (*p* < 0.05). Interrogation of the impact to *icaA* revealed AMI 124A and AMI 133 upregulated the expression by 1.6 to 2.2-folds, but compounds AMI 82B and AMI 129A downregulated it by 0.6 and 0.7-folds, respectively. Only AMI 124A seemed to upregulate *codY* by 1.7-fold (*p* < 0.05). For *alrS*, all of the tested compounds failed to have any significant impact, but was reduced by AMI 133 and AMI 82B by 50 and 30%, respectively. Both *fnbA* and *fnbB* experienced similar responses to the compounds. While AMI 124A downregulated both the genes by ∼0.3-fold, AMI 133 and AMI 82B elevated expression. AMI133 increased *fnbA* and *fnbB* by 1.3 and 1.2-folds, respectively. Similarly, AMI 82B increased expression of both *fnb* genes by ∼1.2-fold. By contrast, AMI 129A did not affect expression levels of *fnb* genes. AMI 124A downregulated the expression of *sarA* by 0.3-fold while AMI 133 and AMI 82B increased expression by ∼1.4 and 1.5-folds, respectively, but once again, AMI 129A did not have an effect. Our findings suggest AMI 82B had the greatest impact with significant reductions in QS genes, decreasing expression of *agr*, *spa*, and *icaA*

## Discussion

QS influences bacterial virulence through expression of virulence factors, and hence, QS antagonists that interfere with bacterial communications are often regarded as a strategy with an overall anti-virulence effect (Salam and Quave, 2018; Piewngam et al., 2020). In the current study, we tested the anti-virulence potential for a panel of substituted diphenyl amide compounds against a community-associated *S. aureus* drug-resistant strain. Overall, the compounds provided insights into efficacy at reducing bacterial virulence with AMI 82B emerging as a hit from the assessed panel.

Our current set of compounds have an excellent anti-QS *IC*_50_ value against *V. harveyi* ranging from 1.1 to 169 μM (Meschwitz et al., 2019). Toxicity assessment in a whole-body animal model revealed varying toxicity profiles even with similar core structures. Interestingly, in the *G*. *mellonella* model, AMI 82A, AMI 82B, AMI 117B, AMI 124A, AMI 129A, AMI 133, and AMII 17C showed very low toxicity profiles (Figure 2). This set of compounds were further evaluated for their therapeutic potential in the same *G. mellonella* during an *S. aureus* infection. For therapeutic response, AMI 82B was found to be the most potent and exhibited 50% larvae survival at 120 and 96 h for 0.5 and 5 mg/kg, respectively. When delivered as a prophylaxis agent, compounds like AMI 117B and AMI 133 were shown to protect 57.5 and 62.5% of the larvae, respectively, at 120 h (*p* < 0.0001) at a low dose of 0.5 mg/Kg.

The compounds were further tested for their hemolytic and cytotoxic behavior. Interestingly, at the highest concentration of 64 μg/mL, none of the compounds were observed to induce hemolysis. Similarly, for the same set of the compounds, the *LD*_50_ on liver-derived the HepG2 cell line was noted to be >64 μg/mL. A low cytotoxicity profile is often needed for any drug-like molecule intended for systemic delivery and the data so far is encouraging for lead compounds.

Anti-biofilm potential was assessed on biomass reduction and live-cell killing in biofilms. There was no reduction in biofilms live cell contents. However, AMI 129A and AMI 133A were able to significantly reduce biomass at 64 μg/mL. Supporting these observations, UM-C162, a benzimidazole derivative, also rescued the nematode from *S. aureus* and prevented biofilm formation without interfering with bacterial viability (Kong et al., 2018). Although our collection of compounds did not impact biofilm as a result of altered QS, some other compounds that affect QS can be seen to influence biofilm formation. RIP (Ribonucleic-acid-III-inhibiting peptide) inhibits staphylococcal TRAP/*agr* systems and has shown biofilm reduction in mice models (Balaban et al., 2007). 2-[(Methylamino)methyl]phenol which targets SarA inhibits biofilm and down-regulates virulence genes (Balamurugan et al., 2017).

To decode influences on genetic contributors to virulence, quantitative PCR was applied. Interestingly, among the tested compounds, AMI 124A and AMI 82B significantly down-regulated the *agr* genes. The *agr* system is involved in regulating the significant virulence genes downstream constituting exo-toxins and exo-enzymes (Recsei et al., 1986). Chemical inhibitors like bumetanide attenuate virulence in *S. aureus* by selectively targeting the AgrA regulator (Palaniappan et al., 2021).

Among our tested compounds, only AMI 82B was responsible for the down-regulation of *spa* gene. The surface protein, *spa* (protein A) is *agr* controlled (RNAIII), and the impact of AMI 82B was not unnatural (Cheung et al., 2011). Spa is an immunoglobulin binding protein involved in both innate and adaptive responses to invasive *S. aureus.* Spa binds B cell receptors, causing B cell death and bringing down the production of *S. aureus*-specific antibodies. We also included an intercellular adhesion gene (*icaA*), responsible for biofilm formation in *Staphylococcus epidermidis* and *S. aureus* (Cramton et al., 1999). We observed mixed regulations of the compounds. While two compounds (AMI 124A and AMI 133) upregulated the expression, the AMI 82B and AMI 129A down-regulated. *S. aureus* biofilm inhibitors like CCG-203592 have been reported to down-regulate *icaA* (Ma et al., 2012). AMI133 and AMI 82B suppressed the *alrS* gene. This cluster of ArlRS is required for virulence in the host. It is reported to be involved in the autolysis and cell surface two-component system, MgrA expression and repression of *agr* (Jenul and Horswill, 2019). The fibronectin-binding proteins A and B (*fnbA* and *fnbB*) were down-regulated by AMI 124A while AMI133 and AMI 82B upregulated them. These are essential adhesins for *S. aureus* infection and biofilm formation (Shinji et al., 2011). A similar expression profiles was observed for *sarA*.

Overall, AMI 82B has emerged as a lead anti-virulence compound that prolongs *G*. *mellonella* survival during infection and appears to upregulate *fnbA* and *fnbB* while reducing *alrS* and *spa* expression. Although AMI 82 B appears to influence virulence, there is no impact directly on bacteria growth or biofilm formation. Since QS antagonists influence bacterial virulence, it is not unusual to retain normal growth rates. Indeed, Gutierrez et al. (2009) demonstrated that three transition state analogs were capable of inhibiting 5′-methylthioadenosine/S-adenosylhomocysteine nucleosidase (MTAN), an autoinducer involved in QS that induce pathogenesis factors in a dose dependent manner but did not influence *Vibrio cholerae* growth. Plant compounds were also found to interfere with QS by Ahmed et al. (2019) who found that *trans*-cinnamaldehyde (CA) inhibits *P*. *aeruginosa* without bactericidal effects. Application of CA reduced expression of QS regulatory genes *lasR* and *rhlR*. The impact was further demonstrated by Rajkumari et al. (2018) who showed CA virulence factors were impacted without affecting bacterial viability. The same report also provided *in vivo* efficacy using a *C*. *elegans* model whereby nematodes experienced prolonged survival from a *P*. *aeruginosa* infection upon treatment with CA, extending survival by 58.63% (Rajkumari et al., 2018). Chen et al. (2019) summarizes findings that include over 100 marine derived QS inhibitors and their synthetic analogs. Within their report, they review QS inhibition by depsipeptides solonamide A and B derived from *Photobacterium* which inhibit *agr* activity in *S*. *aureus* therefore repressing virulence factors. Analogs derived from solonamide B were even more effective than the parent compound at inhibiting AgrC. Here too, the isolated parent solonomide A and B compounds did not affect *S*. *aureus* growth rates in liquid culture (Mansson et al., 2011).

Since lead compounds did not alter bacterial growth rates or exert direct antimicrobial activity, our working hypothesis is that *G. mellonella* prolonged survival is an outcome of reduced virulence. In the presence of the QS antagonist, the virulence of the bacteria might have been impacted and the host immune system was compelling enough to prolong larval survival.

In conclusion, this study presents a series of streamlined experiments to develop anti-QS compounds for antivirulence strategy. Though all the compounds share the same core structure, they present a spectrum of influence on prolonging *G*. *mellonella* survival within the infection model. AMI 82B was the most significant compound of note, prolonging *G*. *mellonella* survival with low toxicity effects to erythrocytes or hepatocytes. Further, AMI 82 presents an interesting compound by reducing expression of three genes (*arg*, *spa*, and *icaA*) and therefore influencing *S*. *aureus* virulence. AMI 82 did not kill *S*. *aureus* cells or reduce growth rates, again suggesting a specific impact on virulence.

## Data Availability Statement

The original contributions presented in the study are included in the article/Supplementary Material, further inquiries can be directed to the corresponding author.

## Author Contributions

SM and MF synthesized compounds and contributed to *in vitro* assessments. BM, RK, and LF conducted *in vitro* assays and *Galleria* infection model assays. SM, EM, and BF designed the experiments. Writing was a joint effort between the authors lead by BM, SM, and BF. All authors contributed to the article and approved the submitted version.

## Conflict of Interest

The authors declare that the research was conducted in the absence of any commercial or financial relationships that could be construed as a potential conflict of interest.

## Publisher’s Note

All claims expressed in this article are solely those of the authors and do not necessarily represent those of their affiliated organizations, or those of the publisher, the editors and the reviewers. Any product that may be evaluated in this article, or claim that may be made by its manufacturer, is not guaranteed or endorsed by the publisher.
