# Supplementary material for: A Substituted Diphenyl Amide Based Novel Scaffold Inhibits Staphylococcus aureus Virulence in a Galleria mellonella Infection Model
Source: Front Microbiol. 2021 Oct 5;12:723133. doi: 10.3389/fmicb.2021.723133 (PMC8524085; doi:10.3389/fmicb.2021.723133)

Figure S1. Whole larvae survival assay for toxicity assessment of additional test compounds. Legend represents: ; No treatment: ; PBS treated: ; DMSO treated: ; Vancomycin treated at 25 mg/Kg: representative compound treated at 0.5 mg/Kg: 5 mg /Kg: 15 mg/Kg and 25 mg/Kg.

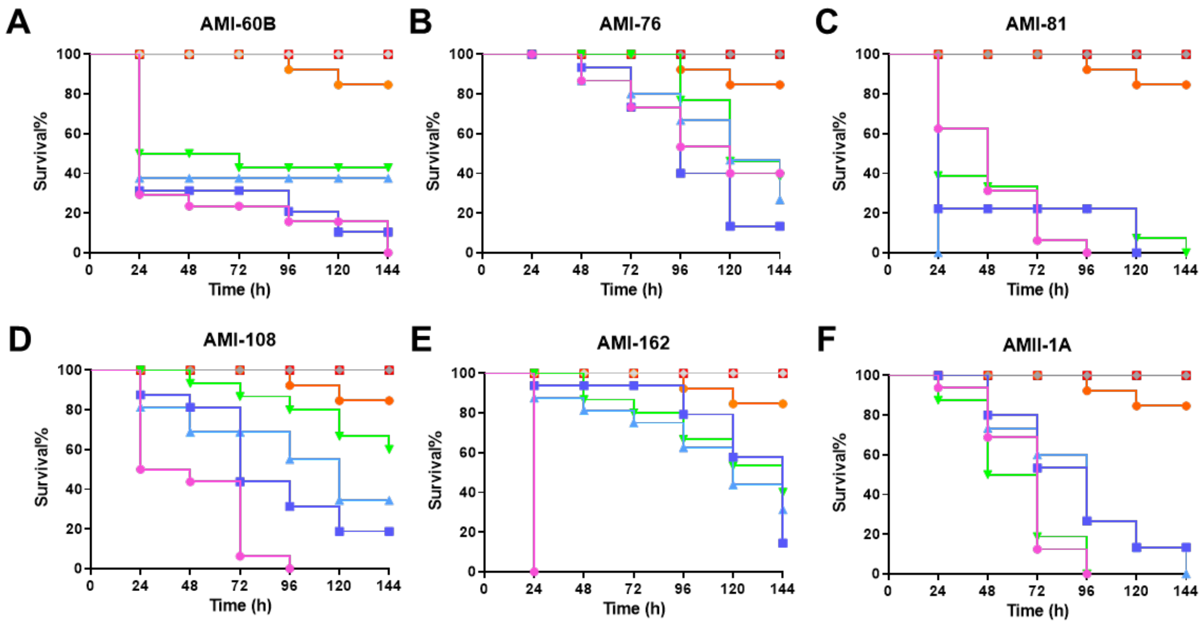

Supplement: Supplementary file 1 [file Image_1.pdf]
