# Supplementary material for: A Substituted Diphenyl Amide Based Novel Scaffold Inhibits Staphylococcus aureus Virulence in a Galleria mellonella Infection Model
Source: Front Microbiol. 2021 Oct 5;12:723133. doi: 10.3389/fmicb.2021.723133 (PMC8524085; doi:10.3389/fmicb.2021.723133)

Figure S2. Growth curves of *S. aureus* MW2 in presence of the lead investigational compounds at 64 μg/ml concentration.

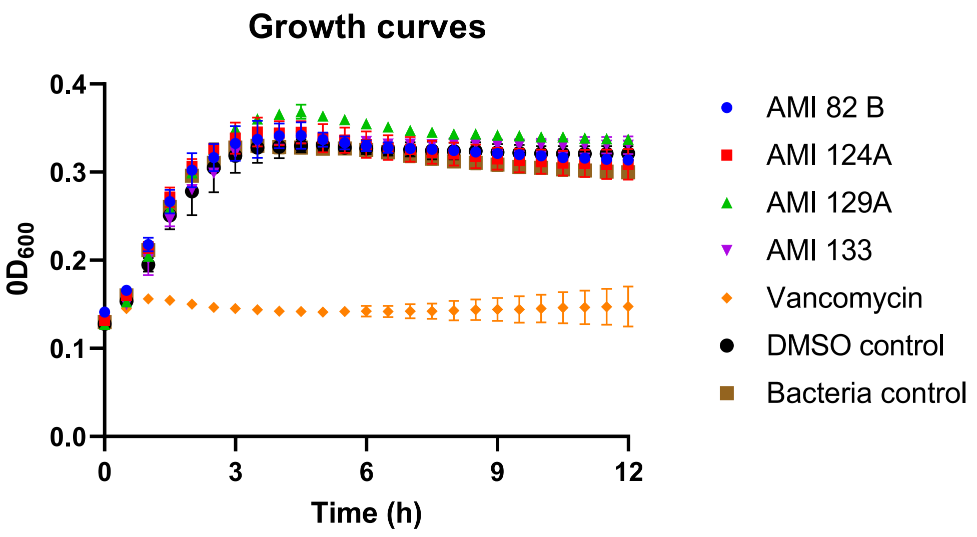

Supplement: Supplementary file 2 [file Image_2.pdf]
