# Supplementary material for: A Substituted Diphenyl Amide Based Novel Scaffold Inhibits Staphylococcus aureus Virulence in a Galleria mellonella Infection Model
Source: Front Microbiol. 2021 Oct 5;12:723133. doi: 10.3389/fmicb.2021.723133 (PMC8524085; doi:10.3389/fmicb.2021.723133)

Figure S3. Effect of lead compounds on live cell inhibition (determined by XTT) of *S. aureus* biofilms at 64 μg/ml concentration.

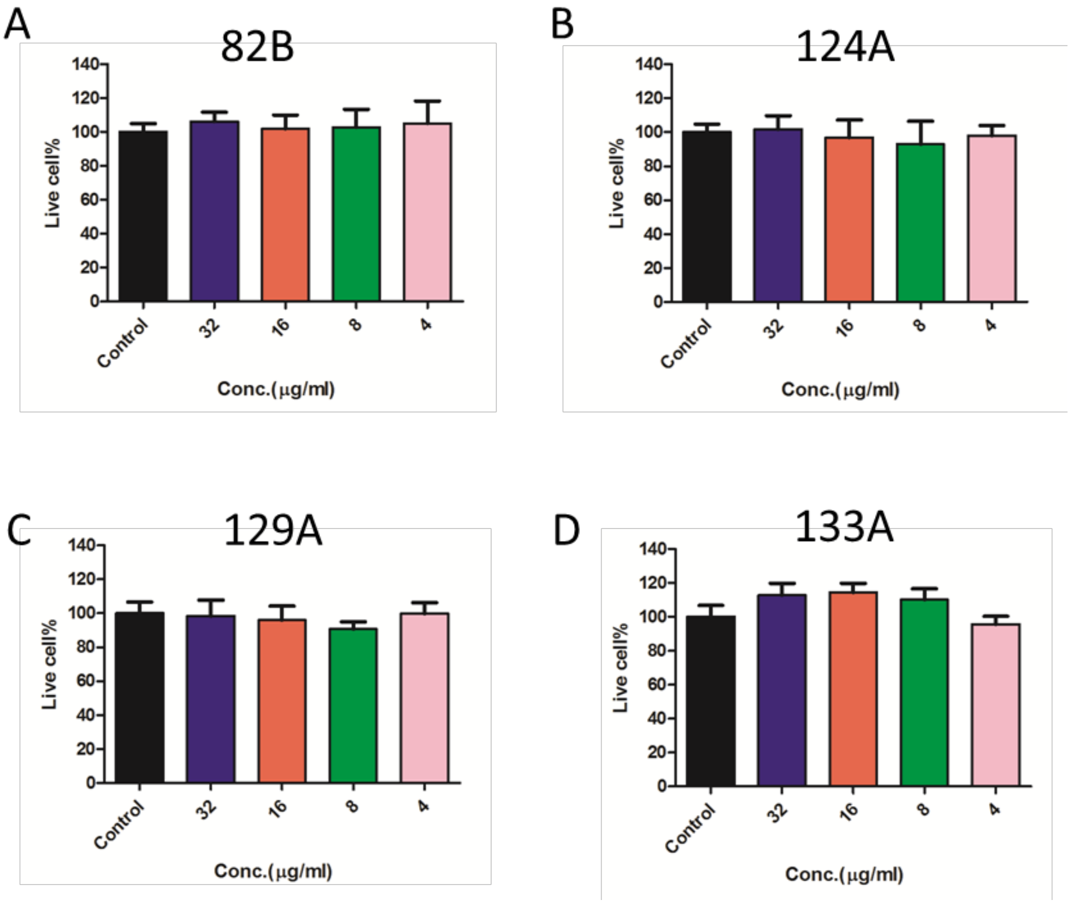

Supplement: Supplementary file 3 [file Image_3.pdf]
