# Supplementary material for: A Substituted Diphenyl Amide Based Novel Scaffold Inhibits Staphylococcus aureus Virulence in a Galleria mellonella Infection Model
Source: Front Microbiol. 2021 Oct 5;12:723133. doi: 10.3389/fmicb.2021.723133 (PMC8524085; doi:10.3389/fmicb.2021.723133)
Supplement: Supplementary file 4 [file Table_1.pdf]

Table S1. Primers for *S. aureus* QS genes

Gene	Role/ description	Sequence	Expected DNA fragment size (bp)
agrA	*AgrA* is the master transcription activator of the *agr* operon. This operon regulates the expression of many toxins, exoenzymes and colonization factors associated with quorum sensing and virulence in *S. aureus* (Le and Otto, 2015).	F: 5′-TGATAATCCTTATGAGGTGCTT-3′<br>R: 5′-CACTGTGACTCGTAACGAAAA-3′	164
arlS	The two-component ArlS-ArlR system is responsible for modifying the activity of extracellular serine protease, autolysis, biofilm formation, capsule synthesis and virulence regulation (Fournier and Rapoport, 2001; Cosby et al., 2020).	F: 5′-TGGAATACCAATTCCATGATCT-3′<br>R: 5′-TGCAATCAAATATGATGTGAAGAA-3′	103
spa	SpA promotes immune invasion by binding to the Fc and Fab region of antibody and B-cell receptor. SpA thus blocks the opsonophagocytosis (Kobayashi and DeLeo, 2013).	F: 5′ -GCGCAACACGATGAAGCTCAACAA -3′<br>R: 5′-ACGTTAGCACTTTGGCTTGGATCA-3′	125
fnbA	Fibronectin binding protein A and B are microbial surface components recognizing adhesive matrix molecules. These are important adhesin (cell wall anchored proteins) that promotes adherence to host tissues in majority of *S. aureus* and is encoded by closely related genes. (Speziale and Pietrocola, 2020; Shinji et al., 2011).	F: 5′-ACTTGATTTTGTGTAGCCTTTTT-3′<br>R: 5′-GAAGAAGCACCAAAAGCAGTA-3′	185
fnbB	Fibronectin binding protein B is known to be involved in binding with histones resulting in neutralization of the antimicrobial activity (Rice et al., 2001; Speziale and Pietrocola, 2020).	F: 5′-CGTTATTTGTAGTTGTTTGTGTT-3′<br>R: 5′- TGGAATGGGACAAGAAAAAGAA-3′	119
icaA	*icaA* encodes for a N-acetylglucosaminyltransferase that synthetizes polysaccharide intercellular adhesin (PIA) oligomers	F: 5′-AACAGAGGTAAAGCCAACGCACTC-3′<br>R: 5′-CGATAGTATCTGCATCCAAGCAC-3′	85

	from UDP-N-acetylglucosamine. (Aricola et al., 2015).		
codY	CodY acts as a repressor to genes involved in nitrogen metabolism. It also regulated *agr* dependent virulence factors (Pohl et al., 2009).	F: 5′-AAAGAAGCGCGCGATAAAGCTG-3′<br>R: 5′-TGCGATTAATAGGCCTTCCGTACC-3′	120
sarA	SarA affects the mRNA turnover of virulence factors protein A (spa) and collagen-binding protein (cna), therefore it is involved in a post-transcriptional regulation of *S. aureus* gene expression (Morrison et al., 2012).	F: 5′-CCTCGCAACTGATAATCCTTATG-3′<br>R: 5′-ACGAATTTCACTGCCTAATTTGA-3′	127
